# Edible agents with perceptible minds: A psychological study of human perception in human–food interaction

**DOI:** 10.1371/journal.pone.0350612

**Published:** 2026-06-22

**Authors:** Takuma Shimoyama, Yuya Kume, Mei Yamagata, Hideyuki Takahashi, Yoshihiro Nakata

**Affiliations:** 1 Graduate School of Informatics and Engineering, The University of Electro-Communications, Chofu, Tokyo, Japan; 2 Faculty of Culture and Information Science, Doshisha University, Kyotanabe, Kyoto, Japan; 3 Faculty of Science and Engineering, Otemon Gakuin University, Ibaraki, Osaka, Japan; Prince of Songkla University, THAILAND

## Abstract

Investigating the psychological and cognitive mechanisms of eating living beings is important for understanding ethical judgments and decision-making in eating behaviors. For example, the meat paradox describes a conflict about harming animals for food. In addition, perceiving a mind in a target has been shown to influence ethical judgment. However, experimentally investigating such food-related ethical themes is difficult, particularly when using actual animals, due to ethical constraints. Therefore, this study proposes an experimental framework using edible agents. Previous research developed a movable edible robot and examined the psychological and cognitive effects of eating moving food. Building on this, we developed an edible agent capable of social interaction through vocalization and evaluated its psychological effects. We examined how people perceive the minds of edible agents with different vocalization types and behaviors, and how this perception influences reluctance to eat and guilt. In the experiment, participants (*N* = 1,094) viewed two videos in which edible agents exhibited different vocalizations and movements and evaluated their impressions. Factor analysis of 18 mind-perception items extracted two factors—Agency and Experience—with a cumulative contribution of 57.8% (α=0.941 and 0.862, respectively). Agency scores were significantly higher in Video 1 than in Video 2 (*W* = 481,686, *p* < .001, *r* = 0.800), whereas Experience scores were higher in Video 2 (*W* = 75,619, *p* < .001, *r* = 0.717). Reluctance to eat differed significantly (*W* = 32,018, *p* < .001, *r* = 0.270), whereas guilt showed no significant difference (*W* = 23,369, *p* = .103, *r* = 0.108). However, no significant relationship was observed between mind perception and either reluctance to eat or guilt. This study provides new insights into social interactions between humans and edible artificial objects, highlighting the potential of edible agents to deepen understanding of food culture and its ethical dimensions.

## Introduction

As concerns grow over a potential shortage of animal-based protein due to the increasing global population [1], it is important to understand the acceptance of food-related cultures, alternative food sources such as insects and frogs, and novel foods enabled by technological advancements. In the acceptance of food cultures and emerging food sources, not only objective indicators such as nutritional value but also human psychological acceptance represents a critical challenge [2]. Psychological barriers to food acceptance include the meat paradox, a moral conflict inherent in consuming animals [3]; disgust theory, which explains the avoidance of contaminated food [4]; food neophobia, defined as the reluctance to consume novel foods [5]; and changes in consumption behavior induced by the anthropomorphism of food [6]. These psychological barriers are considered to arise from prior beliefs and impressions about a target [7]. Among these factors, perceiving a mind in a target is known to influence moral judgment [8], and its relationship with meat consumption has also been suggested [9]. If it becomes possible to flexibly and intentionally manipulate impressions about a target, there is potential to investigate and influence the relationship between humans and food. In this study, we propose an edible agent as a novel experimental tool for investigating such food-related issues by technologically controlling human perception of food.

In a previous study, Nakata et al. introduced the concept of human–edible robot interaction (HERI) and reported that providing autonomous movement to an edible artificial object could make people feel guilty about eating it even though the object was designed to be eaten [10]. This finding suggests that psychological responses similar to ethical conflicts faced by people when they eat animals can also occur with artificial objects. Hence, a new method is required for experimentally investigating psychological and cognitive issues related to meat consumption using artificial objects. The HERI approach offers a realistic and ethically viable alternative for examining morally sensitive issues related to animal life. In animal-based experiments, it is difficult to control variations in the appearance and behavior of the animals, and ethical constraints often limit the experimental design. In contrast, edible robots can be intentionally designed and controlled in terms of appearance, movement, and vocalizations, enabling the quantitative evaluation of human ethical judgments and psychological responses under well-controlled conditions. HERI has attempted to expand the applicability of edible robotics studies [11–13], which focus on applications such as environmental sustainability, medicine, and food transportation, by introducing the aspect of human–robot interaction (HRI). However, there remains a lack of understanding about the human perception of food that exhibits reactive and autonomous behaviors [14].

Human–food interaction (HFI) [15], which is a subdomain of human–computer interaction (HCI), is related to HERI. Previous studies in this field have aimed to enhance food experience through sensory augmentation using taste-based interfaces [16] or by using food as a medium for information transmission, such as edible displays [17] and QR codes [18]. Deng et al. proposed the concept of computational food, wherein food behavior is controlled using computational techniques. They explored HFI using logic bonbons [19,20], which manipulate flavor through fluidic logic circuits, and Dancing Delicacies [21,22], which use moving droplets on a plate. FoodChattAR [23], which uses augmented reality technology to enable a dialogue with food, has been proposed, and social interactions between people and food have been studied in HCI. Based on the aforementioned survey [14] and to the best of our knowledge, no prior study has examined the relationship between mind perception of edible agents and anticipated guilt or reluctance to eat.

In the study by Nakata et al., it was shown that endowing an edible robot with autonomous movement can induce feelings of guilt when consuming it, even though it is an artificial object [10]. Based on this finding, the present study aims to examine the psychological and cognitive factors that elicit ethical responses to the consumption of edible artificial objects. This study focuses on the concept of mind perception. Based on the study by Gray et al. [8], we hypothesize that manipulating the behavior of an edible robot—specifically through differences in vocalization and movement—enables the modulation of two fundamental dimensions of mind perception: Agency (i.e., the capacity for self-control, morality, or intentional action) and Experience (i.e., the ability to experience sensations and emotions such as pain and pleasure). Furthermore, because Experience is closely associated with being a moral patient, we hypothesize that it has a stronger influence than Agency on food-related moral responses, particularly feelings of guilt and reluctance to eat the edible robot. Extensive studies in the field of HRI have examined how people perceive the minds of robots [24,25]; however, robots employed in these studies typically possess mechanical and artificial appearances and are frequently equipped with social cues such as gaze and speech functions. In contrast, the gelatin-based edible robots utilized in the present study exhibit substantial disparities in appearance and texture compared to conventional robots, and it remains uncertain whether people perceive such objects as possessing a mind.

Accordingly, this study aims to examine the proposed hypothesis by developing a socially interactive edible agent based on the movable edible robot developed by Nakata et al. [10]. This agent is capable of engaging in social interactions through vocalizations and movement. The objectives of this study are as follows:

1. To investigate whether people perceive a mind in an edible agent capable of social interaction through vocalizations and movement.
2. To examine how such mind perception influences reluctance to eat and feelings of guilt.

This study was conducted through an online survey without actual eating. This approach was selected because a certain sample size is required to statistically examine whether people perceive a mind in an edible agent, and it is difficult to obtain such a sample size in face-to-face experiments involving actual eating. In addition, examining how mind perception, reluctance to eat, and guilt toward an edible agent vary depending on differences in vocalization and behavior, without actual eating, enables this study to provide insights into impression formation and ethical judgment during the pre-eating stage. This study aims to expand the existing framework of HERI by investigating how socially interactive edible robots affect human cognition and ethical judgment, thereby providing a novel framework to enhance our understanding of food and ethics. This framework has the potential to serve as a new tool for understanding psychological issues related to eating living beings. The key contributions of this study are as follows:

- We developed an edible agent that can engage in social interactions through vocalizations and movement.
- Participants did not actually eat the edible agent; instead, they watched two videos in which the agent engaged in social interaction with a person and evaluated their impressions. These videos demonstrated that mind perception of an edible agent can be modulated. In addition, insights were gained into the relationship between mind perception and anticipated reluctance or guilt associated with eating the edible agent.

The remainder of this manuscript is organized as follows: The research position and potential applications of edible agents are organized, and the structure, control, and operating principles of the edible agent developed in this study are first reviewed. Next, the research questions and hypotheses are presented based on related work. The experimental design and procedures of the online survey are outlined, followed by an explanation of two interaction videos featuring edible agents with different voices and behaviors. The results obtained from the survey are reported, and a discussion is provided on whether mind perception of edible agents can be manipulated and on its relationship with reluctance to eat and feelings of guilt. Finally, the conclusions of this study are presented, along with future directions.

## Edible agent

### Human–edible robot interaction

Interactions between humans and food can be broadly categorized as physical or social. In the context of HFI, physical interactions refer to oral interactions, i.e., sensory experiences that occur in the mouth. This includes how the shape [26] and texture [27] of food affect taste perception and how people respond when eating moving food [10]. Social interactions refer to how people interpret the meaning conveyed by food. These include impressions formed from the arrangement or movement of ingredients [21], psychological responses elicited by eating lifelike artificial objects [10], and psychological reactions to food that exhibit jumping motions triggered by external forces or chemical stimuli [28].

We consider the agency of edible materials in interactions between humans and edible artificial objects. For example, behavior without agency refers to the execution of preprogrammed motions as observed in robotic grippers made from edible materials [11]. In contrast, behavior with agency may involve vibrations that induce a perception of animacy or playback of speech that makes the food appear to be talking. Humans may need to perceive a sense of agency in food to use edible artificial objects as a model for investigating the act of eating living beings.

**Fig 1:**
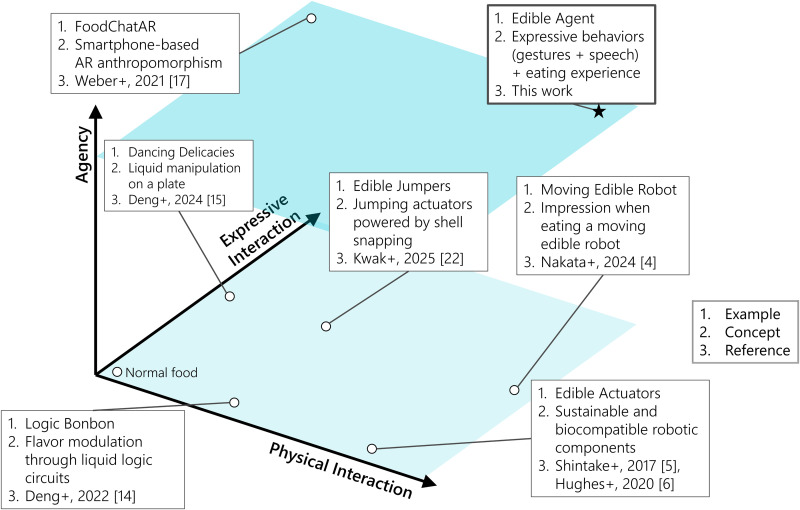
Comparison with previous studies.

Fig 1 summarizes related studies in the fields of HCI (HFI), edible robotics, and HERI in terms of the degree of physical interaction, degree of social interaction, and level of agency. In this study, we implemented a speech function in a previously developed edible robot that can perform physical interactions [10] to achieve more sophisticated social interactions and autonomous behavior.

### Research direction

**Fig 2:**
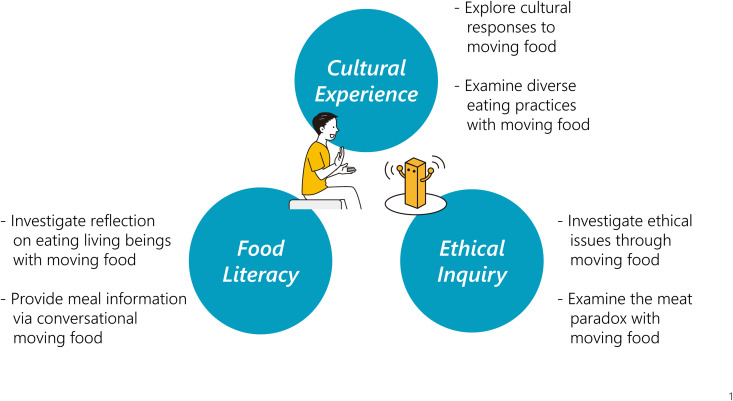
Conceptual diagram for the research direction.

Fig 2 presents a conceptual diagram of the research direction. If an edible agent that can perform autonomous, physical, and social interactions is developed, it can potentially be applied to address the following challenges:

**Cultural Experience:** Animals considered edible vary across countries and cultural spheres, and the ways in which they are consumed are also diverse. For example, in Japan, there is a culinary practice known as “*odorigui*,” wherein live seafood is consumed. Edible agents that can perform physical and social interactions have the potential to offer new insights into food cultures and contribute to mutual cultural understanding.

**Food Literacy:** Edible agents may be utilized in the context of food education. For example, eating a moving edible agent could prompt people to reconsider the meaning of eating living beings. In addition, a conversational edible agent can provide information about the meal itself, potentially fostering a more accurate understanding of the food.

**Ethical Inquiry:** Edible agents may serve as a novel tool for investigating ethical issues related to humans and food. For example, they could offer new experimental insights into issues such as the meat paradox, which is examined in this study.

### Edible-agent development

This section describes the edible-agent system developed in the present study. The system was based on a previously developed edible robot [10]. Gelatin, sugar, calcium carbonate, and 100% apple juice were used to achieve a balance between sufficient mechanical strength to withstand operations and food edibility. For details about the material composition, refer to the previous work [10]. In the present study, several updates were made to the robot to adapt it for use in HERI, which involves social interaction, resulting in the development of a new edible-agent system.

### Structure

**Fig 3:**
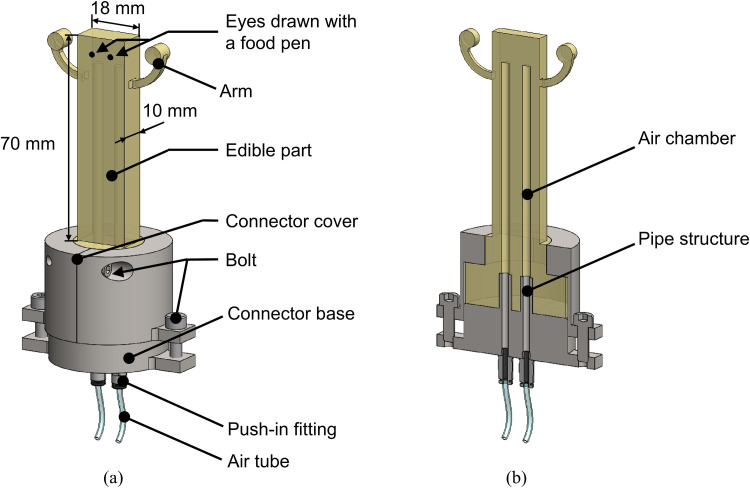
3D-CAD image of the edible agent. **(a)** Overall view. **(b)** Cross-sectional view.

Fig 3 shows three-dimensional computer-aided design (3D-CAD) images of the edible agent, including an overall view (Fig 3(a)) and a cross-sectional view (Fig 3(b)). The edible agent comprises an edible part composed of gelatin and a metallic base component. Two air tubes were connected to the base for supplying air through pipes inserted into the two internal air chambers within the edible part, enabling its motion. The edible part was attached to the pipe of the connector base, connected to the tubes using push-in fittings, and sealed from above with a connector cover. This configuration enabled the edible part to be connected to the tubing without adhesives. These design considerations enable a novel experience in which the edible part can be consumed while it is still moving [10].

In the previous study [10], the edible part of the robot exhibited a simple cuboid shape. In this study, two updates were implemented to perform experiments focused on social interactions. The first update was the addition of arms. Thin arms were added to visually enhance the expression of the movement of the agent, and they oscillated chaotically in response to the swaying of the main edible body, which enabled more dynamic and expressive motion. The second update involved the depiction of the eyes. The eyes play a crucial role not only in human communication [29] but also in interactions with animals such as dogs [30,31]. To encourage people to perceive the edible agent as a communicative entity, eyes were drawn using edible ink with a food-grade pen. In contrast to the prior HERI study [10], which focused on impressions and taste responses to motion, this study introduces vocalizations and expressive movements to enable socially interactive behaviors driven by the edible agent.

**Fig 4:**
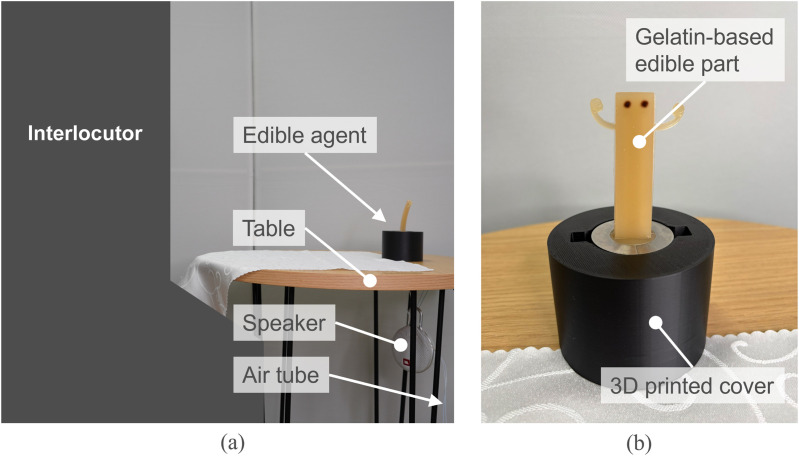
System photographs. **(a)** Recreated setup showing the edible-agent system, with a masked area where an author acted as the interlocutor. **(b)** Edible agent.

Fig 4 shows photographs of the edible-agent system. The edible agent was placed on a table facing a masked area where an author acted as the interlocutor (Fig 4(a)). A speaker installed under the edible agent was used to enable the agent to vocalize. The air tubes were passed through a small hole in the table and connected to a pneumatic circuit. It was possible to eat the edible agent while it remained in motion by lifting a 3D-printed cover (Fig 4(b)).

### Control system

**Fig 5:**
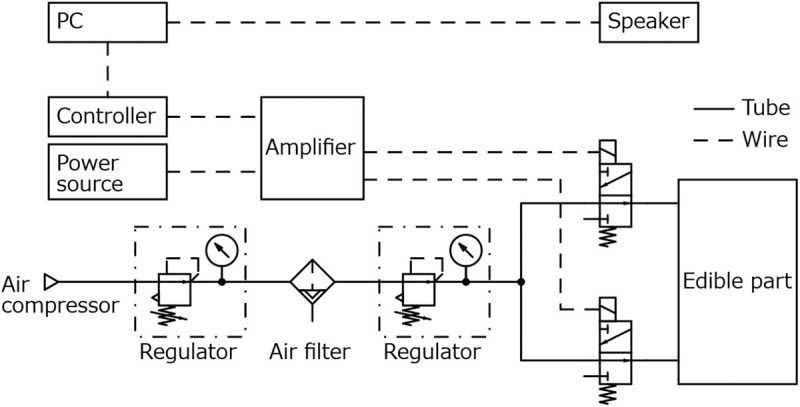
System configuration.

Fig 5 shows the system configuration of the edible agent, and Table 1 lists the devices used in the system. The vocalizations of the agent are played by a personal computer (PC) through the speaker. In addition, the PC sends valve control commands synchronized with the audio files to a microcontroller, which switches the intake and exhaust of the two valves via an amplifier circuit.

**Table 1:** Equipment used in the system.

Device	Model	Manufacturer
Air compressor	SLP-07EED	ANEST IWATA Corp.
Regulator	IR2020-02BG-R	SMC Corp.
Regulator	SRH3001−02	SMC Corp.
Air filter	F8000-20-W-FY	CKD Corp.
Three-port isolated valve	LVMK207-5J	SMC Corp.
Controller	Mbed LPC1768	NXP Semiconductors N.V.
Speaker	JBL Clip 3	Harman International Industries, Inc.

Compressed air is supplied by an oil-free air compressor and regulated to the operating pressure range of the air filter. After passing through the air filter, the compressed air is further regulated to 0.1 MPa and delivered to the edible part via the valves.

### Operating principle

**Fig 6:**
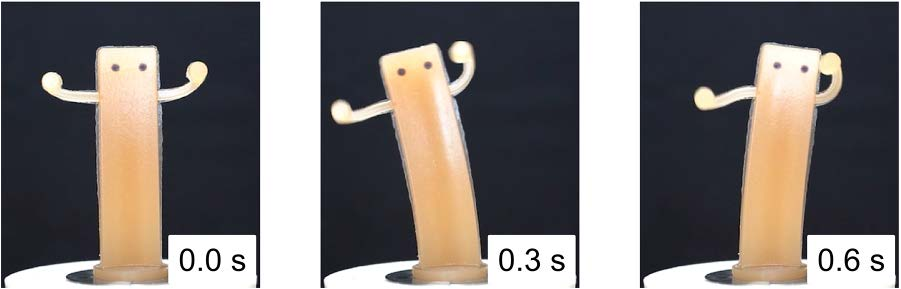
Laterally swinging edible agent.

When compressed air is supplied to one of the air chambers in the edible part, the chamber expands, causing the component to bend in the opposite direction. When equal amounts of air are supplied simultaneously to both chambers, the component extends along the longitudinal axis. A previous study reported that when air was supplied alternately or simultaneously, the edible robot was perceived more highly in terms of perception (emotion, animateness, and intelligence) and taste under an alternating condition [10]. Therefore, in this study, compressed air was supplied alternately to the two air chambers while the agent was speaking, causing the edible agent to swing laterally. Fig 6 illustrates the motion of the edible agent. The air supply and exhaust times were set to 80 and 160 ms, respectively. These durations were empirically determined to produce a swinging motion that appeared natural and synchronized with the speech.

### Related work and research questions

This study investigated the perceptions of edible agents and the relationship between mind perception of the edible agent and the perceived harm of eating it. We examined whether altering the behavior of a gelatin-based edible artificial object affects the degree of mind perception attributed to it, and explored the relationship between mind perception of the edible agent and the perceived harm of eating it. We investigated whether the level of mind perception affects the reluctance to eat the agent or guilt when required to eat it.

### Mind perception of robots

Gray et al. showed that people perceive the minds of characters, both human and non-human, along two dimensions: Agency, which refers to capacities related to self-control and morality, and Experience, which refers to capacities related to sensations such as hunger, fear, pain, and joy [8]. Robots are included among the 13 characters evaluated in terms of mind perception. They are rated lower than adult humans in terms of Agency and Experience, with a particularly pronounced deficit in Experience [8].

Subsequent studies have examined people’s perceptions of robot minds along the dimensions of Agency and Experience. One study showed that robots with human-like appearances were rated higher in terms of Experience than those with mechanical appearances [24]. In contrast, another study reported that robot behavior significantly affected the perceived Agency, whereas appearance had no significant effect [25]. In addition, a separate study evaluating mind perception in both humans and robots suggested that biological features such as a brain or body may be necessary for a robot to be perceived as possessing emotions [32].

### Relationship between mind perception and perceived harm to target

Gray et al. [8] assessed mind perception using a questionnaire that included items on moral judgment. The findings revealed that distress associated with having to harm a character was more strongly correlated with Experience than with Agency. This result suggests that Agency is associated with being a moral agent, whereas Experience is linked to being a moral patient [8].

Even individuals who eat meat may feel distress about harming animals, and this contradiction is referred to as the meat paradox [3,33]. To resolve this cognitive dissonance, people may be inclined to downplay the moral status of animals used as food. For example, it has been reported that individuals evaluate the moral status of cows more negatively after eating beef jerky [3]. In addition, it has been shown that mind perception, evaluated as a single factor, is negatively correlated with perceived edibility of animals and positively correlated with guilt associated with eating them [9].

## Research questions and hypothesis

### Research question 1 (RQ1)

#### Is it possible to manipulate the mind perception of edible agents?.

The appearance and behavior of robots influence mind perception; however, this relationship is not straightforward. If both appearance and behavior contribute to mind perception, it is even less clear whether people can perceive a mind in gelatin-based edible agents such as those used in this study. Clarifying whether people can perceive different types of minds in edible agents would be highly valuable for advancing understanding of HERI.

#### Hypothesis 1 (H1).

It is possible to manipulate the mind perception of edible agents by altering vocalization types and behavior.

To address RQ1 and H1, we prepared two videos depicting edible agents interacting with humans and evaluated mind perception using a within-subject design. In this study, moderate anthropomorphism enhanced the perception of animacy. In Video 1, the edible agent was shown to verbally converse with the participants. In Video 2, the agent responded to the participant’s speech and actions using vocalizations reminiscent of a baby. Through factor analysis of 18 questionnaire items, Agency and Experience were extracted, and their factor scores were calculated. Hypothesis H1 was tested by examining whether there were significant differences in factor scores.

New research directions for leveraging social HERI could be explored if people can perceive different types of minds in socially interactive edible agents. Individuals negatively evaluate the moral status and mind of animals when resolving cognitive dissonance associated with the meat paradox. Similarly, in the context of robots, people attribute greater emotional capacity to robots described as fulfilling social roles (e.g., providing companionship) than to those fulfilling economic roles (e.g., working in a store), and they report lower intentions to harm socially valuable robots [34]. Based on these prior studies, we propose the following research question to address the meat paradox by drawing an analogy between harming robots and eating animals using edible agents.

### Research question 2 (RQ2)

#### Does mind perception influence anticipated reluctance or guilt when eating edible agents?.

The guilt experienced when harming a character or eating an animal may correspond to the guilt associated with eating an edible agent. As an example of HERI research utilizing socially interactive edible agents, this study addresses the meat paradox.

#### Hypothesis 2 (H2).

Mind perception affects anticipated reluctance to eat or guilt associated with eating edible agents, with Experience having a stronger effect than Agency.

To address RQ2 and H2, the participants evaluated their anticipated reluctance to eat or guilt associated with eating edible agents in each video after completing a questionnaire on mind perception. This evaluation was conducted during the same experimental session in which the questionnaire was used to test RQ1 and H1. We examined whether there were significant differences in reluctance to eat and guilt between the video conditions. In addition, we calculated the correlation coefficients between these variables and the dimensions of Agency and Experience and compared their magnitudes. Based on these analyses, H2 was tested.

### Experiment

Securing a sufficient number of participants to conduct factor analysis is necessary because it is not obvious whether people perceive a mind in edible agents. Therefore, instead of conducting an actual consumption experiment, this study employed an online questionnaire in which participants compared two videos using a within-subject design. To enable a direct comparison of mind perception between Videos 1 and 2 and to ensure a common factor structure across conditions, a within-subject design was adopted.

### Participants

An online questionnaire was administered via Yahoo! Crowdsourcing. On the crowdsourcing platform, the survey title and compensation were presented, and participants voluntarily chose to participate in the study. The compensation for completing the survey was 10 JPY. Participation was limited to Japanese individuals aged 20 years or older, as the videos included references to alcohol consumption and were presented entirely in Japanese. A total of 1,484 individuals completed the form. Those who indicated that they were not Japanese nationals were excluded, as were respondents who failed to correctly input keywords presented after each of the five video viewings or failed either of the two directed question scale (DQS) items [35], which were used to detect inattentive responses. Further, when multiple responses were received from the same internet protocol address, all responses were invalidated. Moreover, incomplete responses that were interrupted during submission were invalidated. As a result, 1,094 participants were retained (837 male, 250 female, 7 unspecified; mean age = 55.59 years, range = 20–87 years, SD = 11.50). The age and gender distribution of the participants may have been influenced by the demographics of the crowdsourcing platform.

## Materials and methods

The study protocol was approved by the Ethics Committee of the University of Electro-Communications (No. H24054). The recruitment period for this study was from February 3 to February 4, 2025. Prior to participation, detailed information about the study was provided in the questionnaire form, and informed consent was obtained digitally from all participants.

### Edible agent video conditions

In this experiment, we employed two videos. Video 1 was designed to elicit high ratings for Agency and low ratings for Experience, whereas Video 2 was designed to elicit low ratings for Agency and high ratings for Experience. Gray et al. evaluated mind perception across 13 types of characters, among which God was rated high in Agency and low in Experience, whereas a baby was rated low in Agency and high in Experience [8]. Based on this finding, we created two videos according to the following conceptual framework.

**Fig 7:**
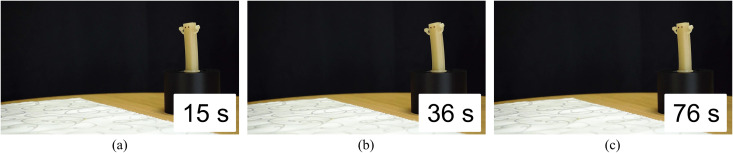
Outline of Video 1. The story depicts an edible agent providing consultation to a human. The first 15 s contain an audio confirmation announcement. The timestamps in the lower right corners of each frame are not present in the original video. **(a)** Initial state at the beginning of the video. **(b)** Agent sways from side to side while speaking. **(c)** Agent remains stationary when not speaking.

Video 1, which was designed to elicit high Agency ratings and low Experience ratings, portrays a scenario inspired by the image of a god or a strict mentor. In this video, a person consults an edible agent regarding personal concerns, and the agent responds rationally without emotional involvement. Fig 7(a)–(c) show an outline of Video 1. Only the edible agent appears on the screen, and the person seeking advice is located off the screen. The edible agent responds in Japanese, swaying side-to-side while speaking, and it remains still when not speaking. The voice was generated using the speech synthesis software Ondoku3 and features a low-pitched male synthetic voice. The total duration of the video was 91 s, with the first 15 s used for audio announcements to confirm proper sound playback.

**Fig 8:**
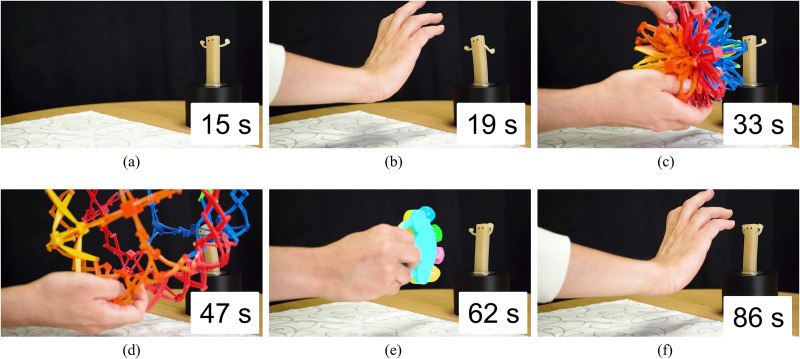
Outline of Video 2. The story depicts an edible agent reacting to a human hand and toys. The first 15 s contain an audio confirmation announcement. The timestamps in the lower right corners of each frame are not present in the original video. **(a)** Initial state at the beginning of the video. **(b)** Agent displays joy in response to a waving hand. **(c)** Agent exhibits fear when confronted with an inflating toy. **(d)** Agent shows anger when the toy inflates repeatedly. **(e)** Agent expresses joy in response to a bell. **(f)** Agent appears sad when the person waves goodbye.

Video 2, which was designed to elicit low Agency ratings and high Experience ratings, was inspired by the image of a baby. In this scenario, when a person waves a hand or shows a toy, the edible agent displays emotional reactions such as joy, fear, and anger. In contrast to Video 1, the edible agent in Video 2 does not engage in verbal conversation but responds with vocalizations resembling the voice of a baby. The audio was created by modifying royalty-free baby sounds to incorporate a slight synthetic quality, ensuring consistency with the conditions in Video 1. Fig 8(a)–(f) show an outline of Video 2. After reacting joyfully to a waving hand (Fig 8(b)), the agent exhibits a fearful response to an expanding toy (Fig 8(c)). When the toy is expanded repeatedly, the agent responds with aggressive movements and an angry-sounding voice (Fig 8(d)). Subsequently, when the bell is shaken, the agent reacts cheerfully and moves in synchrony with the sound (Fig 8(e)). Finally, the agent emits a sad vocalization when the person waves goodbye (Fig 8(f)). The human conversation partner in the video verbalizes the emotions of the agent to aid participant comprehension (e.g., by asking, “Were you surprised?”) because it may be difficult to fully convey emotional changes using vocalizations alone. The total duration of the video was 93 s, with the first 15 s used for audio announcements to confirm appropriate sound playback.

The scripts for each video are provided in the S1 Appendix and S2 Appendix.

### Questionnaire items

To evaluate mind perception of edible agents, participants rated 18 mental capacities used in the study by Gray et al. [8] on a five-point Likert scale (1 = not at all capable, 3 = moderately capable, 5 = extremely capable). The items presented to the participants were obtained from the Japanese version of the mind perception questionnaire developed by Kamide et al. [36]. The questions were as follows.

To what extent do you think the edible agent shown in Video X is capable of the following?

- Communication
- Consciousness
- Desire
- Embarrassment
- Emotion recognition
- Fear
- Hunger
- Joy
- Memory
- Morality
- Pain
- Personality
- Planning
- Pleasure
- Pride
- Rage
- Self–control
- Thought

In addition, two questions regarding personal judgments of edible agents were asked using a seven-point Likert scale. To account for possible experimenter effects, the wording of the questions did not explicitly include the terms “reluctance to eat” or “guilt.” For example, if participants were asked, “How much do you not want to eat it?,” they could feel compelled to respond in a way that aligns with not wanting to eat. The two questions are as follows.

- If you were required to eat the edible agent, which of the following would best describe your feeling? (1 = strongly want to eat, 4 = neither, 7 = strongly do not want to eat)
- If you were required to eat the edible agent, which of the following would best describe your feeling? (1 = feel no guilt at all, 4 = neither, 7 = feel a great deal of guilt)

### Procedure

**Fig 9:**
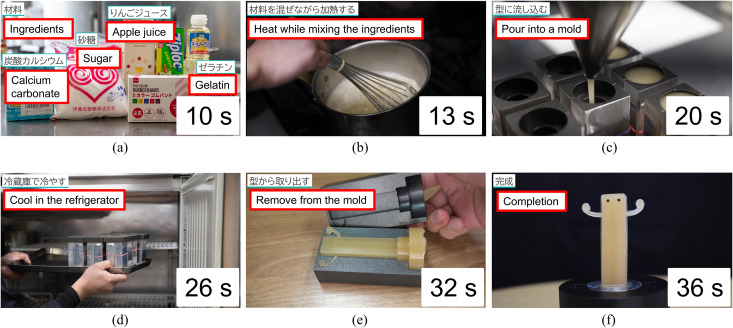
Outline of the video generation process for the edible agent. The first few seconds display a notice indicating that this video has no audio. The red-framed sections contain English translations of Japanese captions, which are not included in the original video. The timestamps in the lower right corners of each frame are also not present in the original video. **(a)** Ingredients. **(b)** Mixing the ingredients while heating. **(c)** Pouring the mixture into a mold. **(d)** Cooling the mixture in a refrigerator. **(e)** Removing the edible part from the mold. **(f)** Completion.

Participants first watched a 42-s video that showed how the edible part of the agent was made. This was intended to emphasize that the agent was made from familiar edible ingredients such as gelatin, sugar, and apple juice, reinforcing the impression that it could be eaten. An outline of the video preparation is shown in Figs 9(a)–(f). Next, the participants reported their sex, age, and nationality and then watched Videos 1 and 2 consecutively. Subsequently, they rewatched Video 1 and rated the edible agent in that video on 18 mental capacities. The same procedure was followed for Video 2. Finally, the participants rated their anticipated reluctance to eat and guilt associated with eating for Videos 1 and 2.

The presentation order of Videos 1 and 2 was randomized across participants to counterbalance potential order effects and mitigate demand characteristics or response bias. A unique keyword was displayed at the end of each of the five video views to confirm that the participants viewed the videos. The participants were required to select the correct keyword from a list of options. In addition, the order of the 18 items assessing mental capacity was randomized. A DQS item reading “Please do not select any answer for this question” was always presented in the fixed 13^th^ position to help identify inattentive responses.

The links to the videos are provided in the S3 Appendix.

### Data analysis

First, factor analysis was conducted on the 18 items assessing the mind perception of the edible agent. In this study, the scenarios of the videos were designed with reference to characters such as God and a baby, which were identified by Gray et al. [8] as having extreme profiles along the dimensions of mind perception. To facilitate comparison of the perceived minds of the edible agents with the findings of Gray et al., the number of factors was set to two. Subsequent analyses used promax rotation and a regression method.

Subsequently, Wilcoxon signed-rank tests were conducted with the two extracted factors as dependent variables to investigate the effects of video content on mind perception. Wilcoxon signed-rank tests were also conducted for anticipated reluctance to eat and guilt. Steiger’s Z-test was used to compare differences between correlation coefficients for examining the relationship between mind perception and reluctance to eat or guilt. For all statistical analyses conducted in this study, the significance level was set at α=0.050.

## Results

The two factors extracted through factor analysis had a cumulative contribution of 57.8%. As shown in Table 2, factor 1 represents the Agency of the edible agent (α=0.941), and factor 2 represents Experience (α=0.862).

**Table 2:** Factor loadings of mental capacities.

	Factor	
	1	2
**Agency (α=0.941)**		
Thought	**0.915**	−0.114
Morality	**0.904**	−0.183
Planning	**0.884**	−0.177
Self–control	**0.857**	−0.141
Pride	**0.801**	−0.010
Memory	**0.781**	−0.023
Consciousness	**0.710**	0.174
Communication	**0.673**	0.094
Desire	**0.644**	0.174
Emotion recognition	**0.577**	0.256
Personality	**0.576**	0.222
**Experience (α=0.862)**		
Rage	−0.165	**0.886**
Fear	−0.156	**0.839**
Pain	−0.065	**0.777**
Pleasure	0.000	**0.692**
Embarrassment	−0.005	**0.673**
Joy	0.061	**0.634**
Hunger	0.175	**0.375**

**Fig 10:**
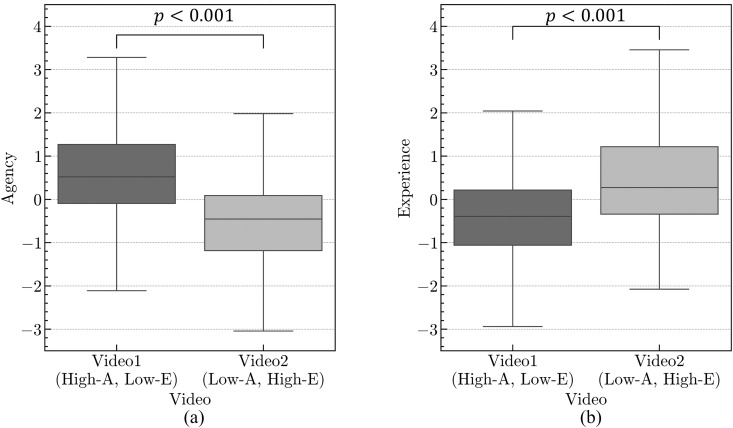
Box plots of factor scores for mind perception. (a) Agency. (b) Experience. The central line indicates the median, the box represents the interquartile range (IQR), and the whiskers extend over the range of the data. The vertical axis represents factor scores derived from the factor analysis, which are dimensionless.

For mind perception of the edible agent, the Agency score for Video 1 (*Mdn* = 0.520) was significantly higher than that for Video 2 (Mdn=−0.453, *W* = 481,686, *p* < .001, *r* = 0.800), with a location shift of 0.978 (95% CI [0.887, 1.072]), as shown in Fig 10(a). Further, as shown in Fig 10(b), the Experience score for Video 2 (*Mdn* = 0.272) was significantly higher than that for Video 1 (Mdn=−0.395, *W* = 75,619, *p* < .001, *r* = 0.717), with a location shift of −0.779 (95% CI [−0.861,−0.699]).

**Fig 11:**
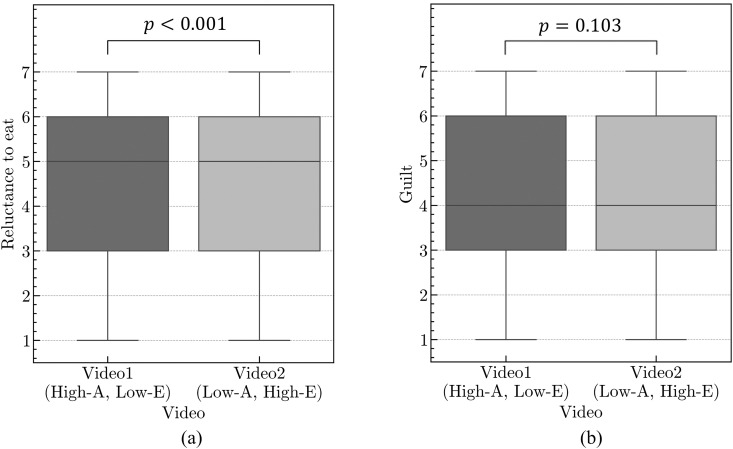
Box plots of personal judgments. (a) Reluctance to eat. (b) Guilt. The central line indicates the median, the box represents the IQR, and the whiskers extend over the range of the data. The vertical axis represents Likert-scale scores (1–7), which are dimensionless subjective ratings.

For anticipated reluctance to eat, reluctance to eat the edible agent in Video 1 (*Mdn* = 5.000) was significantly higher than that for Video 2 (*Mdn* = 5.000, *W* = 32,018, *p* < .001, *r* = 0.270), with a location shift of 0.500 (95% CI [0.000, 1.000]), as shown in Fig 11(a). For guilt, no significant difference was observed between the edible agents in Video 1 (*Mdn* = 4.000) and Video 2 (*Mdn* = 4.000, *W* = 23,369, *p* = .103, *r* = 0.108), with a location shift of 0.000 (95% CI [−0.000, 0.500]), as shown in Fig 11(b).

For the relationship between mind perception and reluctance to eat or guilt, no significant difference was found between the Spearman correlations of reluctance to eat with Agency (*r* = 0.089) and Experience (*r* = 0.085, *z* = 0.110, *p* = 0.913). Similarly, no significant difference was observed between the Spearman correlations of guilt with Agency (*r* = 0.138) and Experience (r=0.185, z=−1.313, p=0.189).

## Discussion

This study experimentally investigated whether altering the vocalization types and behaviors could change the mind perception of gelatin-based edible agents. Furthermore, it examined whether mind perception of edible agents influences anticipated reluctance to eat or guilt associated with eating them. Edible artificial objects that can perform both physical and social interactions with humans have not been realized previously. The proposed system may serve as a novel tool for experimentally investigating challenges related to humans eating living beings.

In the experiment conducted to test H1, we investigated whether altering the vocalization types and behaviors of edible agents would change mind perception. Through an online survey, participants evaluated their perceptions of edible agents in two videos featuring interactions with humans. Video 1 showed an edible agent responding to the consultation of a person in Japanese. The video was designed to elicit high Agency and low Experience ratings. Video 2 depicted an edible agent reacting to the person’s hand and toys, responding with baby-like vocalizations. The video was designed to elicit low Agency and high Experience ratings. The results indicated that the Agency score for Video 1 (High-A, Low-E) was significantly higher than that for Video 2 (Low-A, High-E). Conversely, the Experience score for Video 2 was significantly higher than that for Video 1. These findings support H1 and demonstrate that mind-perception evaluations of the two types of edible agents designed to evoke images of God/strict mentors and babies, respectively, are consistent with those reported by Gray et al. This suggests that people can perceive distinct minds even for simple gelatin-based edible artificial objects. In addition, these results provide the first evidence that humans perceive different minds in edible artificial agents and imply that the proposed edible agent can serve as a useful tool for investigating issues related to humans eating living beings.

In the same experiment, the anticipated reluctance to eat and guilt associated with eating edible agents were evaluated (RQ2) to test H2. Reluctance to eat was significantly higher in Video 1 (High-A, Low-E) than in Video 2 (Low-A, High-E). However, there was no difference in the medians (both *Mdn* = 5.000), and the effect size was relatively small (*r* = 0.270), suggesting only a modest difference despite statistical significance. No significant differences were observed for guilt. Next, Spearman correlations were calculated to examine the relationship between mind perception and reluctance to eat or guilt, and the differences were tested. No significant differences were found between the correlations of reluctance with Agency and Experience, or among guilt, Agency, and Experience. Therefore, Hypothesis H2 was not supported. These results indicate that although mind perception was robustly manipulated, its influence on food-related moral responses was limited under the present experimental conditions. This pattern should be interpreted in light of broader literature on eating-related moral responses. Prior studies on meat consumption suggest that negative responses to eating animals are influenced not only by mind attribution but also by multiple psychological factors, including disgust and cognitive processes related to moral conflict. Disgust has been identified as a fundamental mechanism underlying food rejection, particularly in the context of potentially harmful or undesirable food stimuli [4]. In addition, food neophobia has been shown to reduce willingness to consume unfamiliar foods [5], which may be relevant given the novelty of the edible agents used in this study. Furthermore, meat consumption is associated with cognitive dissonance, often referred to as the meat paradox, in which individuals experience conflict between caring for animals and consuming them [3]. To resolve this conflict, people may reduce the perceived moral status or mental capacities of animals, including the denial of mind [9]. Such processes can attenuate moral concern and may limit the emergence of guilt, even when a target is perceived as having a mind. From this perspective, the absence of a significant effect on guilt in the present study does not necessarily contradict prior findings on mind perception. Rather, it suggests that the relationship between mind attribution and moral emotions in eating contexts may be modulated by additional factors such as disgust, novelty, and cognitive dissonance.

These evaluations addressed the meat paradox using an edible agent. Although the relationship between mind perception and the reluctance to eat or guilt was not elucidated, the study is valuable in that it presents a novel experimental framework to investigate ethical issues underlying meat consumption using an edible agent.

## Conclusion

In this study, we developed edible agents with eyes, arms, and vocalizations to enhance their perceived animacy and to investigate psychological responses to food under controlled conditions. The results demonstrated that mind perception can be systematically manipulated through differences in behavior and vocalization (RQ1). However, its influence on food-related moral responses was limited, with only a modest effect on reluctance to eat and no significant relationship with guilt (RQ2). A potential limitation is that both human speech and baby-like vocalizations may have evoked a sense of humanness. Additionally, because a video-based experiment was employed to ensure sufficient participant evaluation, reluctance to eat and guilt were assessed without actual consumption. The edible agents did not possess advanced autonomy. Future studies should investigate how autonomous behaviors during actual consumption—such as resistance behaviors when placed in the mouth—and the incorporation of sensing and feedback mechanisms influence human responses. Furthermore, it will be important to examine the effects of varying the number of agents and introducing multi-agent interactions to clarify how perceived relationships among agents affect reluctance to eat and guilt. The findings obtained in this study establish edible agents as a controllable experimental framework for examining psychological and ethical aspects of eating that are difficult to study using real animals, and for understanding how perception relates to food acceptance in the context of food-related ethical conflicts, while highlighting the importance of extending this framework to more realistic conditions in future research.

## Supporting information

S1 Appendix Script in Video 1 (High-A, Low-E). (PDF)

S2 Appendix Script in Video 2 (Low-A, High-E). (PDF)

S3 Appendix Links to videos. (PDF)
