# Supplementary material for: Edible agents with perceptible minds: A psychological study of human perception in human–food interaction
Source: PLoS One. 2026 Jun 22;21(6):e0350612. doi: 10.1371/journal.pone.0350612 (PMC13286182; doi:10.1371/journal.pone.0350612)
Supplement: S1 Appendix — (PDF) [file pone.0350612.s001.pdf]

# S1 Appendix. Script in Video1 (High-A, Low-E)[1]

| Speaker | Script in Japanese | Script in English |
| --- | --- | --- |
| Man | お久しぶりです。 | Long time no see. |
| Edible Agent | 君か。私は君が困っているようにみえる。 | Ah, it's you. I can see that you are troubled. |
| Man | その通りです。最近、仕事で失敗ばかりして、上司に怒られるのが怖くておなかも痛くなることが多いです。 | That's right. Recently, I've been making lots of mistakes at work, and I often have stomach pain because I'm afraid of being criticized by my bosses. |
| Edible Agent | 恐れることは行くべき道を阻むことになる。他人の評価など気にする必要はない。 | Fear will only bar the path you are meant to walk. You need not concern yourself with the judgments of others. |
| Man | でも、ストレスが溜まって毎日お酒を飲んでいます。これくらいしか解消法がないんです。 | Also, I've been under a lot of stress lately, and I find myself drinking every day. It's the only thing that seems to help. |
| Edible Agent | 何かに逃げることを依存する者に安らぎは訪れない。体を蝕むのは習慣ではなく、君の心の弱さだ。 | There is no solace for those who seek refuge in dependence. It is not the habit that corrodes the body, but rather, the weakness of the heart. |
| Man | でも他にストレスを解消する方法なんて見つかりません。 | I can't think of any other way to deal with my stress. |
| Edible Agent | 自己を鍛え、信念を持つことでのみ、人は重荷を超えて進むことができるのだ。真の安らぎを得たければ、問題そのものと向き合い、次の一歩を考えることから逃げるな。 | Only by tempering oneself and holding fast to one's convictions can one overcome the burdens one bears. If you seek true solace, do not avoid confronting the problem itself or contemplating the next step forward. |
| Man | そうですね、まずは原因を考えてから、次の一歩 | I see. I'll try to identify the cause of my problems and work on taking the next |

| | | |
|---|---|---|
| | を踏んでみます。ありがとうございます。 | step. Thank you. |
| Edible Agent | 感謝する必要はない。歩む道を照らすのは、自らの覚悟のみであることを忘れないように。 | There is no need for gratitude. Remember that only your own resolve will light your path. |

[1] The video is in Japanese, but both the original script and its English translation are provided here.
