# Supplementary material for: Edible agents with perceptible minds: A psychological study of human perception in human–food interaction
Source: PLoS One. 2026 Jun 22;21(6):e0350612. doi: 10.1371/journal.pone.0350612 (PMC13286182; doi:10.1371/journal.pone.0350612)
Supplement: S2 Appendix — (PDF) [file pone.0350612.s002.pdf]

S2 Appendix. Script in Video2 (Low-A, High-E)[2]

Speaker	Script in Japanese	Script in English
Man	久しぶり。	Long time no see.
Edible Agent	（嬉しい声）	(Happy voice)
Man	元気にしてそうだね．今日はおもちゃを持ってきたんだ。ちょっと待ってね。まず一つ目はこのおもちゃだよ。このおもちゃ知っている？	You look like you've been doing well. I brought some toys today. Just a moment... The first one is this toy. Do you know what this is?
Edible Agent	...	...
Man	まあ、わからないよね。このおもちゃは膨らんだり、しぼんだりするんだ。	Well, I suppose you wouldn't know. This toy can expand and condense.
Edible Agent	（怖がる声）	(Frightened voice)
Man	びっくりした？ほらほら見て！これすごいよね？！	Did I surprise you? Look! Isn't this amazing?!
Edible Agent	（怒っている声）	(Angry voice)
Man	ごめんね。怒らせちゃったね。次からは気を付けるよ。もう一つおもちゃがあるんだ。次は気に入ってくれると思うよ。次はこのおもちゃだよ。これは知っている？	Sorry, I made you angry. I'll be more careful from now on. I have another gift for you. I think you'll like this one. This time, it's this toy. Do you know what this is?
Edible Agent	...	...
Man	わからないよね。これはこうやって振ると音が鳴るんだよ．	You didn't know that, did you? Shaking it like this makes a sound.
Edible Agent	（喜んでいる声）	(Joyful voice)
Man	やっぱり音が鳴るものが好きなんだね．喜んでいる姿を見れて嬉しいよ．そしたらそろそろ帰るね．会えてうれしかったよ．またね．バイバイ！	I knew it, you really like things that make sounds! I'm glad to see you so happy. Well then, I should get going. I'm really happy I got to see you! See you again. Bye-bye!

Edible Agent	（寂しそうな声）	(Sad voice)

[2] The video is in Japanese, but both the original script and its English translation are provided here.
