# Supplementary material for: Edible agents with perceptible minds: A psychological study of human perception in human–food interaction
Source: PLoS One. 2026 Jun 22;21(6):e0350612. doi: 10.1371/journal.pone.0350612 (PMC13286182; doi:10.1371/journal.pone.0350612)
Supplement: S3 Appendix — (PDF) [file pone.0350612.s003.pdf]

S3 Appendix. The links to each video

Video 1 (High-A, Low-E)
https://youtu.be/0_hgrf0pi2Y

Video 2 (Low-A, High-E)
https://youtu.be/0GW9b6Sk-9U

Video of how to make the edible agent.
https://youtu.be/S75xd_0SOVs
